# Probucol attenuates high glucose-induced Müller cell damage through enhancing the Nrf2/p62 signaling pathway

**DOI:** 10.1007/s10792-023-02859-z

**Published:** 2023-09-09

**Authors:** Yu-Fan Zhou, Heng-Wei Liu, Xu Yang, Chen-Xiang Li, Jian-Su Chen, Zhong-Ping Chen

**Affiliations:** 1 Department of Ophthalmology, Aier Eye Hospital of Changsha, Changsha, 410015 Hunan Province China; 2 Aier School of Ophthalmology, Central South University, Changsha, 410015 Hunan Province China; 3 Aier Eye Research Laboratory, Aier Eye Institute, Changsha, 410015 Hunan Province China; 4 Medical College, Jinan University, Guangzhou, 510632 Guangdong Province China; 5 The First Clinical Medical College of Jinan University, Guangzhou, 510000 China; 6 School of Stomatology and Ophthalmology, Xianning Medical College, Hubei University of Science and Technology, Xianning, 437100 China

**Keywords:** Probucol, Diabetic retinopathy, Human Müller cells, Nrf2/p62 signaling pathway, Cell cycle, Cell damage

## Abstract

**Purpose:**

This study investigated the protective effect of probucol on Müller cells exposed to high glucose conditions and examined potential mechanisms of action.

**Methods:**

Primary human retinal Müller cells were incubated with high glucose (HG, 35 mM) in the present or absence of different concentrations of probucol for 24 h. Cell viability was determined using the CCK-8 method. Mitochondrial membrane potential (MMP) was measured using JC-1 staining and cell cycle by flow cytometry. The expression of nuclear factor E2-related factor 2 (Nrf2), glutamate–cysteine ligase catalytic subunit, and p62 was quantified using quantitative polymerase chain reaction and western blot.

**Results:**

We found that HG inhibited cell proliferation, arrested cell cycle, and increased MMP in human Müller cells. Probucol activated the Nrf2/p62 pathway and upregulated the anti-apoptotic protein, Bcl2, and attenuated HG-mediated damage in Müller cells.

**Conclusions:**

Our results suggest that probucol may protect Müller cells from HG-induced damage through enhancing the Nrf2/p62 signaling pathway.

## Introduction

Diabetic retinopathy (DR) is one of the most common and serious complications of diabetes mellitus. Currently, there are limited treatments for DR, and none of them can prevent its progression [1]; the outcome of DR depends on early recognition and early intervention. The pathogenesis of DR remains to be fully elucidated although oxidative stress is known to play a critical role [2]. Chronic hyperglycemia leads to the production of reactive oxygen species (ROS) through various pathways [3]. The retina is rich in mitochondria and has a high respiration rate to meet its metabolic needs; thus, the retina is prone to ROS production and is susceptible to oxidative damage. In DR, an imbalance between ROS production and antioxidant defense systems causes retinal oxidative damage and retinal cell death, which contributes to the pathogenesis of DR [4–6].

Müller cell malfunction critically contributes to the development of DR. These cells can release a variety of cytokines and growth factors, some of which are injurious to the retina and are related to vascular dysfunction and generation of new blood vessels, whereas others are beneficial in protecting retinal neurons from various damages; for instance, Nrf2 is mainly expressed in Müller cells in the retina [7–9]. Studies have shown that chronic hyperglycemia can first induce apoptosis in Müller cells and then cause changes in the retinal microenvironment and accelerate neuronal apoptosis, finally leading to the occurrence and development of DR [8, 10]. Normalizing Müller cell function is considered a good therapeutic strategy to prevent or treat DR.

Nuclear factor E2-related factor 2 (Nrf2) is one of the most important endogenous antioxidant pathways. Nrf2 can adjust the expression of antioxidant genes and detoxifying enzymes by reacting with antioxidant components (ARE) (including glutamate cysteine ligase catalytic subunit [GCLC], p62, etc.). GCLC can regulate the synthesis of glutathione and exert antioxidant effects [11]. Autophagy is another pathway that maintains cell homeostasis in vivo under oxidative stress; excessive ROS production can activate both autophagy and the Nrf2 pathway [12, 13]. p62 is a substrate for autophagy and may play a role in maintaining protein homeostasis and clearing damaged proteins through autophagy [14]. Further, p62 can activate the Nrf2 signaling pathway by interacting with Keap1. Nrf2 regulates p62 expression, resulting in positive feedback regulation between the two proteins [15, 16]. Autophagy and the Nrf2 pathway are closely connected via the Nrf2/p62 pathway, which plays a crucial role in maintaining homeostasis [17, 18]. Dysregulation of the Nrf2/p62 pathway has been shown to play an important role in the occurrence and development of various diseases, such as diabetic heart disease [19]. Thus, therapeutic approaches targeting the Nrf2/p62 pathway are expected to be beneficial in DR.

Probucol (PB), an anti-hyperlipidemic, anti-oxidative, and anti-inflammatory drug, has two phenolic hydroxyl groups in its molecular structure that are easily oxidized, exerting its strong antioxidant effect [20]. PB can improve cognitive dysfunction caused by oxidative stress in diabetic rats through Nrf2 pathway activation [21]. Further, PB can induce autophagy, reduce nerve cell apoptosis, and promote neurological function recovery after spinal cord injury, also promote neuroregeneration and ameliorate functional deficits in traumatic brain injury [22, 23]. PB has also been demonstrated to inhibit penile cell apoptosis and autophagy in diabetic rats [24]. Previously, we have shown that PB can inhibit intracellular ROS generation, promote proliferation and decrease Müller cell apoptosis under high glucose (HG) conditions [25]. In this study, we further investigated the mechanism of PB in protecting primary human Müller cells from HG-mediated damage, including the Nrf2/p62 signaling pathway.

## Materials and methods

### Cell culture and identification

The study protocol was approved by the Ethics Committee of AIER Eye Group. All donated human eyeballs were provided by the AIER Eye Bank of Changsha (Changsha, China). Consent forms were obtained by the Eye Bank. 10 eyes were used in this study. Human primary Müller cells were isolated and cultured as described previously [25]. The phenotype of cultured Müller cells was verified by immunolabeling for glutamine synthase (GS).

### Cell proliferation assay

Cell proliferation was assed using the CCK-8 kit (Solarbio, Beijing, China) according to the manufacturer’s instructions. Briefly, cells were cultured in triplicate in 96-well plates (5 × 10^3^ cells/well) in low glucose (LG, 5.5 mM, mimic normal state) or high glucose (HG, 35 mM, mimic diabetic state). Some cells under HG conditions were treated with different concentrations of PB (10 μM, 30 μM, 50 μM, 100 μM, and 150 μM) for 24 h after HG treatment for 24 h. Subsequently, cells were incubated with 10 μl CCK-8 solution at 37 ℃ for 4 h. Optical density values were measured using a multi-functional microplate reader (BioTek, VT, USA) at 490 nm.

### Mitochondrial membrane potential (MMP) analysis

The fluorescent, lipophilic, and cationic probe JC-1 (Beyotime, China) was used to measure MMP according to the manufacturer’s directions. Briefly, Müller cells were seeded in a 12-well plate (1 × 10^5^ cells/cell), which were routinely maintained in LG (low glucose—5.5 mM) media, which was the control. When we started the experiment, we first used HG (high glucose—35 mM) treatment for 24-h to mimic diabetic state (hyperglycemia) in vitro, then we added PB into HG cultured cells for additional 24-h as drug intervention (HG + PB group). Subsequently, the medium was removed, the cells were washed twice with cold phosphate-buffered saline (PBS), and then treated with JC-1 (10 mg/mL, 0.5 mL/well) at 37 °C for 30 min. After washing twice with 1 × incubation buffer, fluorescence intensity was immediately measured by fluorescence microscopy. For JC-1 green, Ex = 485 nm and Em = 525 nm; for JC-1 red, Ex = 535 nm and Em = 590 nm.

### Cell cycle assay

Cell cycle was analyzed using the Cell Cycle and Apoptosis Analysis Kit (Beyotime, China) according to the manufacturer’s instructions. Briefly, Müller cells were seeded in 6-well plates (2 × 10^5^ cells/well). After treatment with LG, HG and HG + PB (as detailed above), the cells were collected and fixed in ice-cold 70% ethanol at 4 °C overnight. On the next day, the cells were washed with PBS and stained with PI in the dark for 30 min at 37 °C. Samples were analyzed using flow cytometry (BD FACSCelesta, USA), and 30,000–80,000 cells were collected per sample. Data were analyzed using FlowJo 10.6 software.

### RNA extraction and quantitative polymerase chain reaction (qPCR)

Total cellular RNA was extracted from different groups of Müller cells using TRIzol Reagent (Thermo Scientific, USA). The cDNA was synthesized using Revert Aid First Strand cDNA Synthesis Kit (Vazyme Biotech, Nanjing, China) according to the manufacturer’s instructions. Gene-specific primers were synthesized by TsingKe Biotech (Wuhan, China). The primer sequences used were as follows: Nrf2 (sense: TCAGCCAGCCCAGCACATCC; antisense: TCTGCGCCAAAAGCTGCATGC), p62 (sense: TCCAGGATCAGGGGTTAGGG; antisense: TAGGCAAGCTATGTGCTGGG), GCLC (sense: ACGGAGGAACAATGTCCGAG; antisense: TACTGAAGCGAGGGTGCTTG), β-actin (sense: CATGTACGTTGCTATCCAGGC, antisense: CTCCTTAATGTCACGCACGAT). Gene expression was then analyzed by real-time PCR (Roche, Switzerland) with 40 cycles of 95 °C for 15 s and 60 °C for 30 s. The β-actin gene was used as an internal control. The mRNA expression levels were calculated using the 2^−△△Ct^ method.

### Western blot (WB) analysis

Müller cells were washed with cold PBS, and total proteins were extracted with RIPA buffer (Beyotime, China) supplemented with a Protease Inhibitor Cocktail (Amyjet Scientific Co., Ltd, Wuhan, China). A BCA protein assay kit (Vazyme Biotech) was used to analyze the protein concentrations of the cell lysates. The protein samples (15 μg every sample) were subjected to sodium dodecyl sulfate-polyacrylamide gel electrophoresis (SDS-PAGE) followed by transfer to polyvinylidene fluoride (PVDF) films. After blocking for 2 h in 5% BSA (w/v), the PVDF membranes were incubated overnight at 4 °C with the following primary antibodies: rabbit anti-Bcl2 (1:1000, Arigo, China); rabbit anti-GCLC (1:1000, Abcam, UK); rabbit anti-p62(1:1000, Abcam, UK); and mouse anti-GAPDH (1:1000, Abcam, UK). The membranes were then rinsed three times and incubated with anti-rabbit/mouse IgG (1:5000, Molecular Probe, USA) for 2 h. An electrochemiluminescence ECL kit (Thermo, USA) was then used to observe the immunoreactive bands on the Odyssey Fc Imaging System (LI-COR Biosciences, USA) followed by quantification using ImageJ software.

### Statistical analysis

SPSS 25.0 and GraphPad Prism 8.0 software were used to analyze the data and plot graphs. Pearson's Chi-square test was used to compare cell cycles between the three groups, other comparisons among the three groups were performed using one-way ANOVA followed by Dunnett’s or Dunnett’s T3 post hoc tests. The results were expressed as the mean ± standard error. All experiments were repeated at least three times. *P* < 0.05 was used to indicate statistical significance.

## Results

### Morphological observation and identification of primary Müller cells

**Fig. 1:**
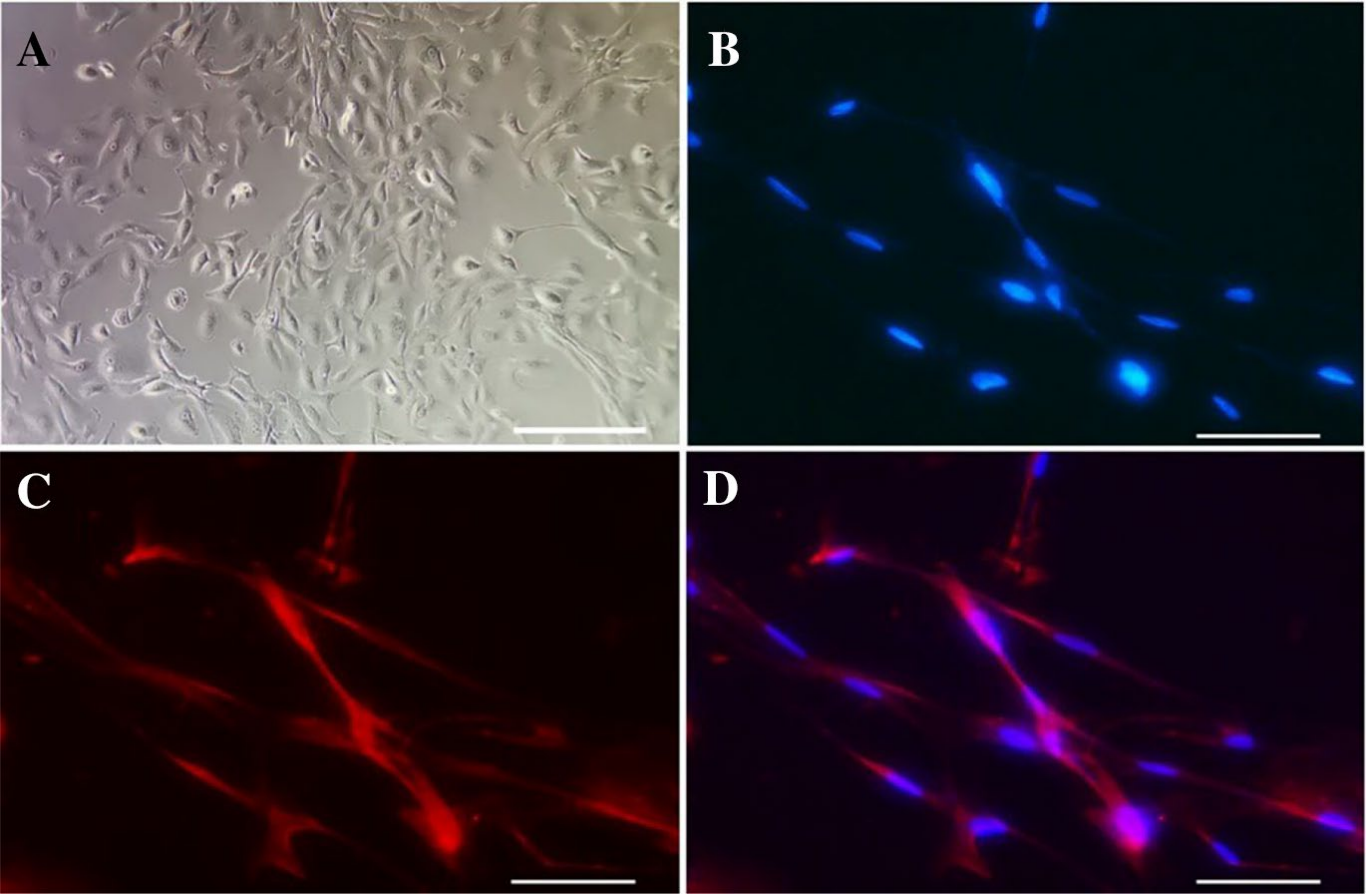
Morphology and identification of human Müller cells. **A** Morphology of primary Müller cells. Scale bar = 30 μm. **B**–**D** Identification of Müller cells by GS immunofluorescence staining. **B** The nuclei of Müller cells were stained with DAPI. **C** GS expression in Müller cells. **D** The merged image of **B**, **C**. Scale bar = 15 μm. *GS* glutamine synthase

The cultured Müller cells at passage 3 were presented as cobblestone-like and some were elongated under phase-contrast light microscope (Fig. 1A). Immunofluorescent investigation showed that > 90% of cells were positive for GS (Fig. 1B–D), indicative of high purity primary human Müller cells. Cells at passages 3–5 were used for this study.

### Protective effect of PB against HG-induced cytotoxicity in Müller cells

**Fig. 2:**
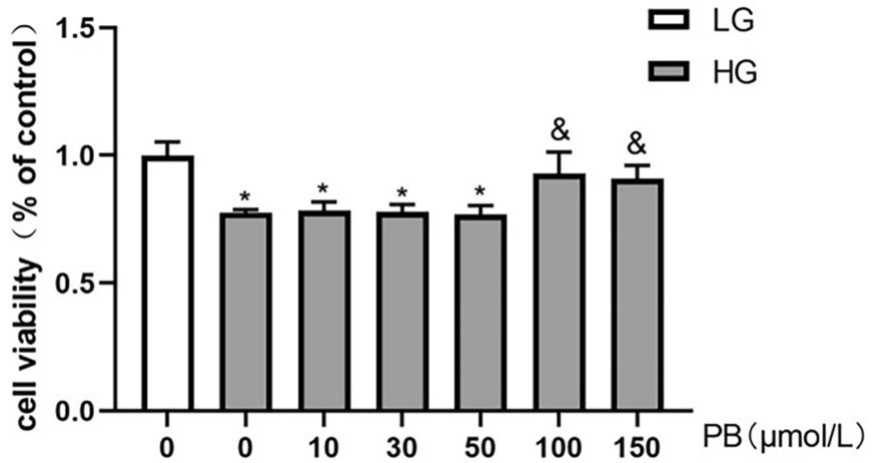
PB improved Müller cell viability under HG conditions. Primary human Müller cells were incubated with 5.5 mM (LG) or 35 mM (HG) glucose for 48 h. The HG treated cells were also treated with or without different concentrations of PB. Cell viability was measured by CCK8 assay. Mean ± SEM, *n* = 3. *P < 0.05 versus the LG group. ^&^*P* > 0.05 versus the LG group. One-way ANOVA followed by Dunnett’s post hoc tests. *HG* high glucose, *LG* low glucose, *PB* probucol

When Müller cells were cultured with 35 mM glucose (HG) for 48 h, the viability was significantly reduced compared to 5.5 mM glucose (LG) treatment (Fig. 2). PB at the concentrations of 100 and 150 μM significantly protected Müller cell from HG-induced cell death. PB at the concentrations of 10–50 μM did not show any protective effect (Fig. 2).

### PB inhibited HG-induced reduction in mitochondrial potential and Bcl2 expression in Müller cells

**Fig. 3:**
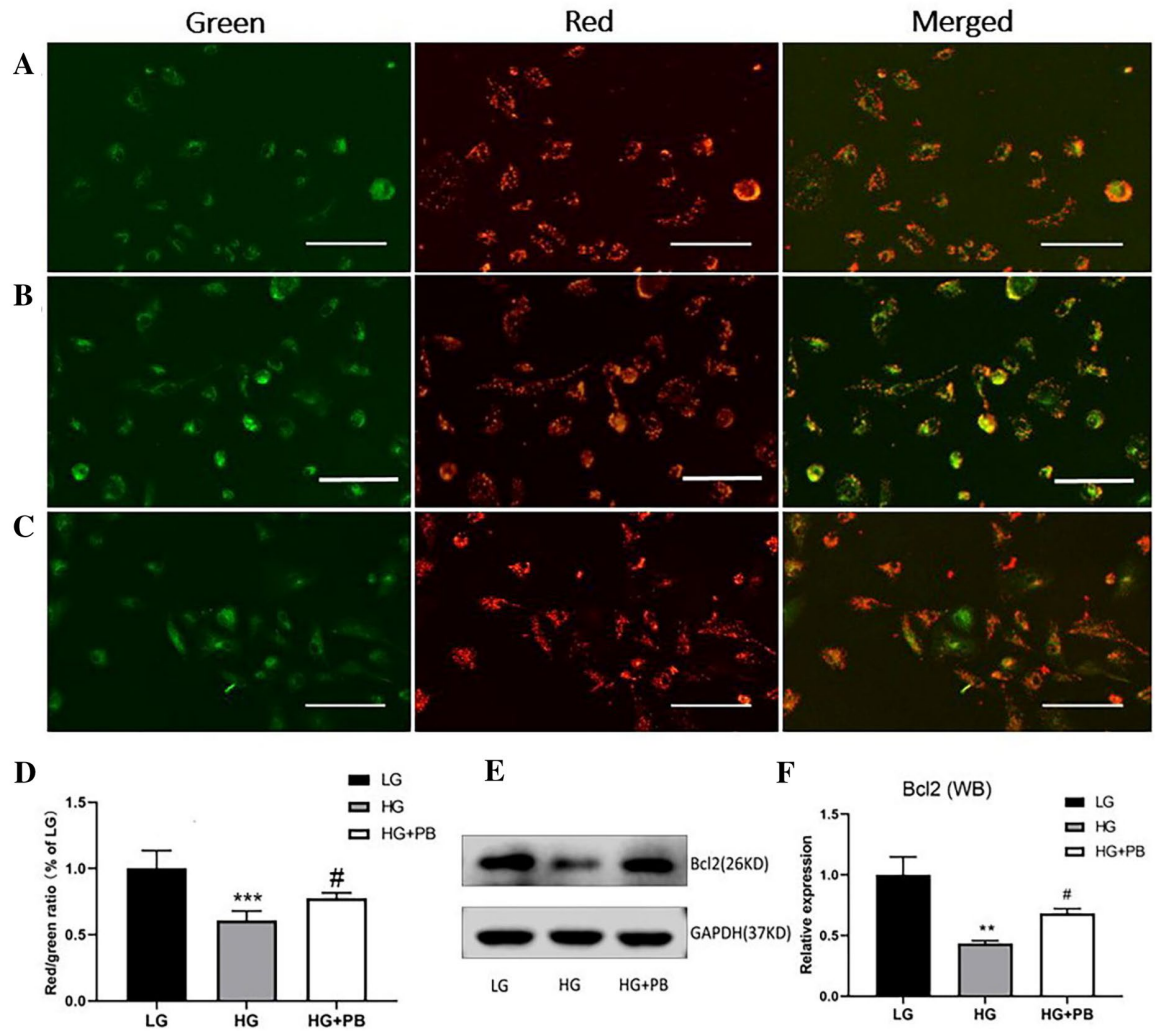
PB reduced HG-induced apoptosis in Müller cells. **A**–**C** Representative images of JC-1 staining of Müller cells in the LG, HG, and HG + PB groups, Green: JC-1 monomer (green), Red: JC-1 polymer. Scale bar = 15 μm. **D** Analysis of fluorescence intensity in Müller cells. Mean ± SEM, *n* = 3. ***P < 0.001 versus the LG group. #*P* < 0.05 versus the HG group. One-way ANOVA followed by Dunnett’s post hoc tests. **E** Representative WB showing the expression of Bcl2 and housekeeping protein GAPDH. **F** Bar graph showing the relative expression of Bcl2. Mean ± SEM, *n* = 3. **P < 0.01 versus the LG group. One-way ANOVA followed by Dunnett’s post hoc tests. ^#^*P* < 0.05 versus the HG group. One-way ANOVA followed by Dunnett’s T3 post hoc tests. *HG* high glucose, *LG* low glucose, *PB* probucol

To further explore the mechanism of protective effect of PB on HG-induced Müller cell death, we examined the mitochondrial membrane potential (MMP) in Müller cells using JC-1 staining along with the analysis of anti-apoptosis protein, Bcl2, using WB analysis. GH treatment significantly reduced MMP in Müller cells (Fig. 3A, B), but the reduction was prevented by PB treatment (100 μM of PB, Fig. 3A–D). The expression level of Bcl2, as shown in Fig. 3E, F, was significantly reduced under the HG condition compared with that in the control under LG conditions. PB treatment prevented HG-induced Bcl2 reduction.

### PB inhibited the HG-mediated S phase arrest in Müller cells

**Fig. 4:**
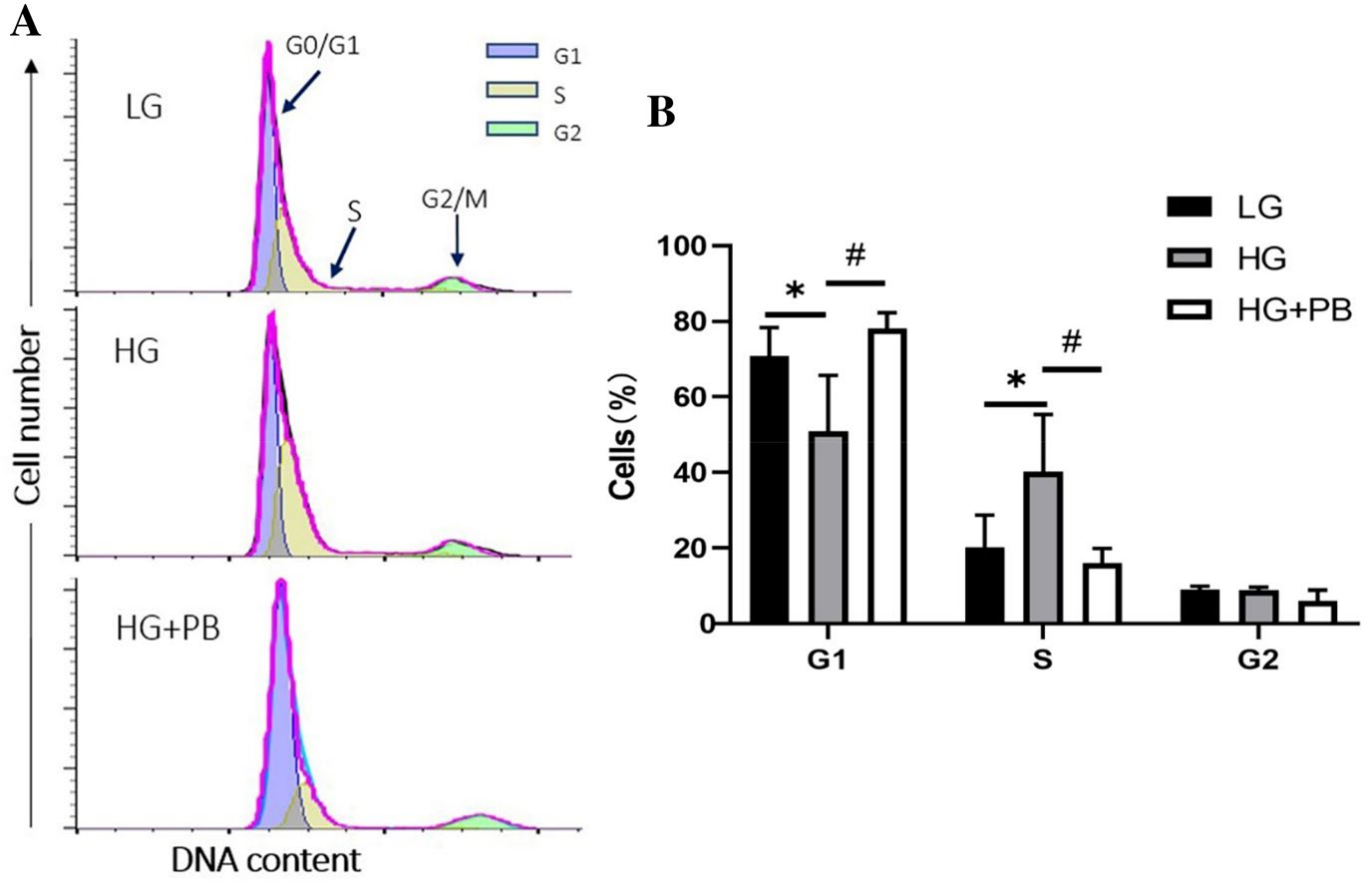
PB prevented HG-induced S phase arrest in Müller cells. **A** Representative plots of cell cycle distribution in the LG, HG, and HG + PB groups. **B** Statistical chart of the proportion of cell cycle phases in each intervention group. Mean ± SEM, *n* = 420,463. *P < 0.05 versus the LG group. ^#^*P* < 0.05 versus the HG group. Pearson's Chi-square test was used. *HG* high glucose, *LG* low glucose, *PB* probucol

We next investigated the effect of PB on HG-induced on the cell cycle arrest. In cells treated with HG, the percentage of cells in G1 phase was reduced and the percentage of cells in the S phase was increased (Fig. 4). PB treatment (100 μM) prevented HG-induced S phase arrest (*P* < 0.05, Fig. 4A, B).

### PB activated the Nrf2/p62 pathway in HG-treated Müller cells

**Fig. 5:**
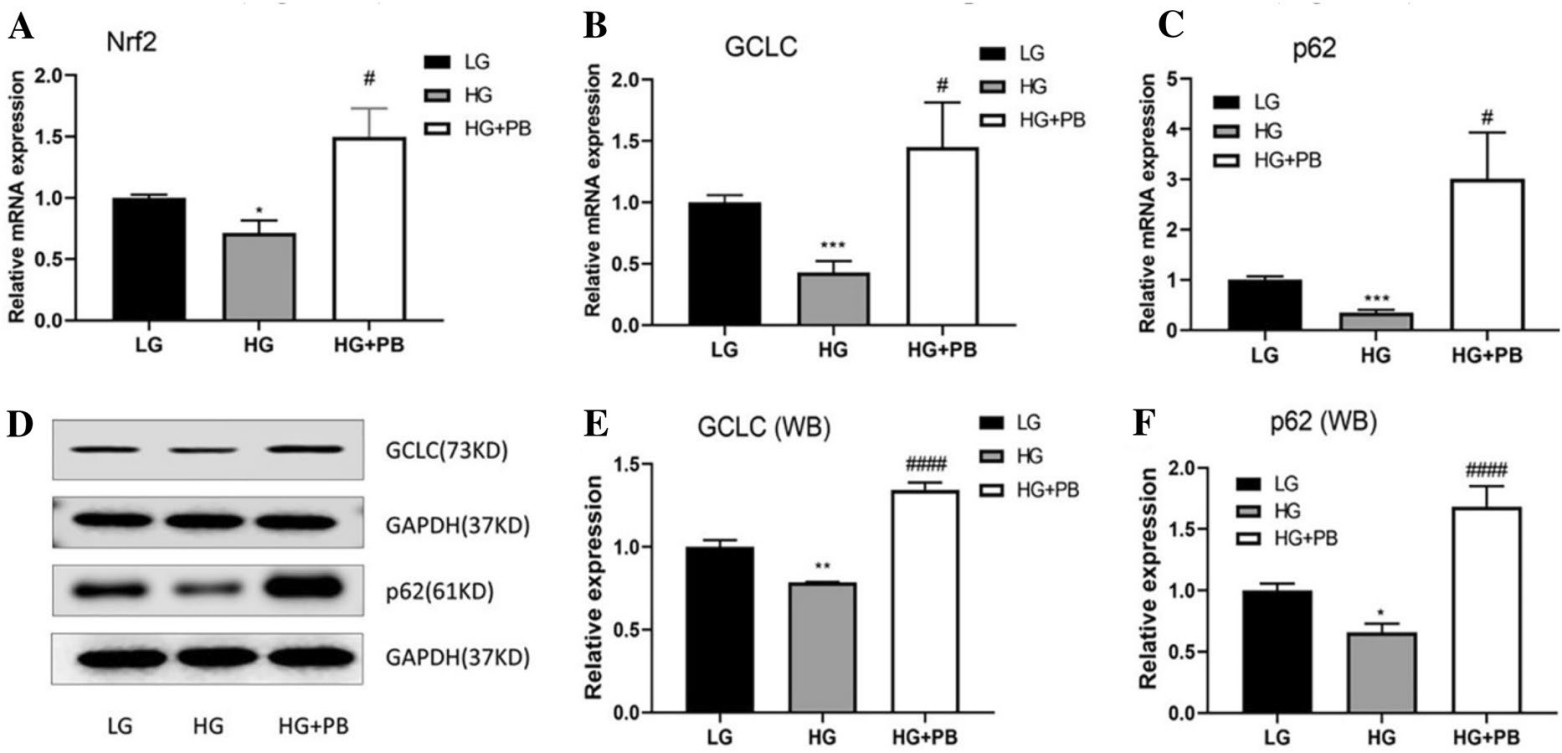
PB activated the Nrf2/p62 antioxidant signaling pathway in Müller cells. **A**–**C** Nrf2, GCLC, and p62 mRNA expression levels. Mean ± SEM, *n* = 12. *P < 0.05 versus the LG group. ***P < 0.0001 versus the LG group. ^#^*P* < 0.05 versus the HG group. One-way ANOVA followed by Dunnett’s T3 post hoc tests. **D**–**F** GCLC and p62 protein expression levels. Mean ± SEM, *n* = 3. *P < 0.05 versus the LG group. **P < 0.01 versus the LG group. ^####^*P* < 0.00001 versus the HG group. One-way ANOVA followed by Dunnett’s post hoc tests. *HG* high glucose, *LG* low glucose, *PB* probucol, *Nrf2* Nuclear factor E2-related factor 2, *GCLC* glutamate cysteine ligase catalytic subunit

To explore the effect of PB on the Nrf2/p62 pathway, the mRNA expression levels of Nrf2, GCLC, and p62 were determined by qPCR, and the protein expression of GCLC and p62 was analyzed by WB. HG treatment significantly reduced the mRNA expression of Nrf2, GCLC and p62 genes (Fig. 5A–C), and this was prevented by PB treatment (Fig. 5A–C). This effect was further confirmed at the protein levels by WB (Fig. 5D–F).

## Discussion

Diabetic retinopathy shows increasing prevalence and incidence rates worldwide, and the risk of vision loss is very high. Currently, various therapeutic methods, including laser therapy, antibody injection, and vitrectomy, have been developed for treatment, which play an important role in preventing vision loss [3]. However, since treatments to restore retinas to pre-diabetic retinopathy states have not yet been developed, inhibiting progression from non-proliferative diabetic retinopathy diabetic retinopathy to proliferative diabetic retinopathy is more important in preventing vision loss and is the subject of intensive research [1]. We discovered a lipid-lowering drug that could prevent the occurrence of diabetic retinopathy in previous studies [19, 25], and we conducted the current study to further evaluate the efficacy of the natural product extracts and their underlying mechanisms.

Müller cells, which are pivotal for maintaining retinal homeostasis, exposure to high glucose levels can lead to retinal damage and dysfunction. Müller cell dysfunction is considered to being a major cause of DR, including diabetic retinal vasculopathy and neuropathy [10, 26], although the underlying mechanism remains poorly defined. In the present study, we found that HG could reduce Müller cell viability through multiple mechanisms, including affecting mitochondrial membrane potential, inducing cell cycle arrest, and powering the expression of anti-apoptotic molecule Bcl2.

PB, a lipid-lowering drug, has strong antioxidant effects due to its unique molecular structure of the phenolic hydroxyl group. The beneficial effects of PB on various chronic diseases have been described previously [21, 22]. Recent evidence indicated that PB has a great therapeutic potential in diabetes and its complications, such as diabetic nephropathy [25]. We found that PB at the concentration of 100 μM and above could protect Müller cells from HG-mediated toxicity. PB treatment increased MMP and Bcl2 expression, normalized cell cycle in Müller cells cultured under HG conditions.

The Nrf2 pathway is the most important endogenous antioxidant pathway and significantly contributes to the regulation of oxidative stress and apoptosis in various cells [27]. Malfunction of the Nrf2 pathway is known to contribute to many neurological diseases, such as Parkinson’s disease and Alzheimer’s disease [28–31]. Notably, this pathway has been reported to be associated with diabetic complications, including diabetic nephropathy [32] and DR [29]. Autophagy has been shown to result in prolonged Nrf2 activation in a p62-dependent manner. p62, an autophagy adaptor and acceptor, has a Keap1-interacting region domain, which allows p62 to sequester Keap1 into autophagosomes and impairs Nrf2 ubiquitylation [33], thus leading to further activation of the Nrf2 signaling pathway [34]. Under normal conditions, the Nrf2-Keap1-p62 loop is in a dynamic steady state to maintain cellular redox homeostasis [15]. In diabetes, the increased ROS production leads to autophagy activation, which prompts p62 to bind with Keap1 and subsequently inactivates Nrf2 [35]. In this study, we found that PB could activate the Nrf2/p62 signaling pathway in Müller cells under HG conditions. The expression of its downstream antioxidant reaction elements GCLC was also normalized by PB treatment. These data suggest that PB might protect Müller cells from HG by activating the Nrf2/p62 pathway.

Given the detrimental impact of high glucose levels on Müller cell function and retinal health, identifying therapeutic agents that can mitigate this damage is essential. Probucol, with its antioxidant and potential autophagy-inducing properties, offers promise as a potential therapeutic option for managing retinal complications associated with high glucose levels. However, at present, it is challenging to foresee the potential clinical application of the findings due to the absence of animal or human data in this study. Nevertheless, ongoing tests are in progress to investigate the in vivo effects of PB treatment.

## Conclusions

In conclusion, this study highlights the beneficial effects of probucol in attenuating high glucose-induced damage to Müller cells, potentially through enhancing the Nrf2/p62 signaling pathway. The findings provide valuable insights into the molecular mechanisms underlying these protective effects and suggest probucol as a potential therapeutic candidate for managing retinal complications associated with high glucose levels. Further research is needed to uncover the precise mechanisms and determine the clinical viability of probucol as a treatment option.

## Data Availability

All data generated or analyzed during this study are included in this published article.

## Abbreviations

HG: High glucose
LG: Low glucose
PB: Probucol
MMP: Mitochondrial membrane potential
Nrf2: Nuclear factor E2-related factor 2
GCLC: Glutamate cysteine ligase catalytic subunit
qPCR: Quantitative polymerase chain reaction
DR: Diabetic retinopathy
ROS: Reactive oxygen species
ARE: Antioxidant components
GS: Glutamine synthase
PBS: Phosphate-buffered saline
WB: Western blot
SDS-PAGE: Sodium dodecyl sulfate-polyacrylamide gel electrophoresis
PVDF: Polyvinylidene fluoride
